# Supplementary material for: An analysis of neuroscience and psychiatry papers published from 2009 and 2019 outlines opportunities for increasing discovery of sex differences
Source: Nat Commun. 2022 Apr 19;13:2137. doi: 10.1038/s41467-022-29903-3 (PMC9018784; doi:10.1038/s41467-022-29903-3)
Supplement: Supplementary file 1 — Supplementary Information [file 41467_2022_29903_MOESM1_ESM.pdf]

Country/Region of Author Affliation	Discipline	Total number of papers: 2009	Total number of papers: 2019	Number of papers that analyzed: 2009	Number of papers that analyzed: 2019
U.S.A.	Neuroscience	631	354	4	15
	Psychiatry	226	186	10	18
Canada	Neuroscience	39	17	0	1
	Psychiatry	11	9	1	1
E.U.	Neuroscience	181	110	2	1
	Psychiatry	89	42	1	1
Asia	Neuroscience	57	67	0	0
	Psychiatry	28	20	2	0
U.K.	Neuroscience	46	30	0	0
	Psychiatry	23	12	2	0
Combination	Neuroscience	390	341	3	10
	Psychiatry	147	118	8	14

Supplementary Table 1. Total number and number of papers by country of author affiliation and discipline and number of papers that had studies that analyzed using sex as a discovery variable by country of author affiliation and discipline. Combination was a combination of countries.

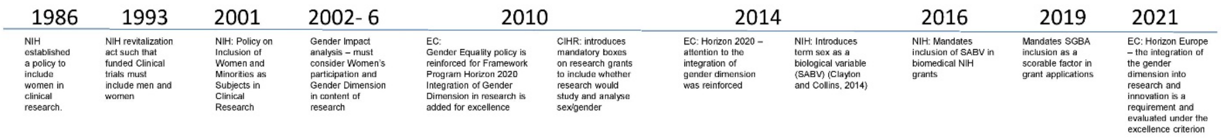

Supplementary Figure 1. Timeline of Mandates across years from the National Institute of Health (NIH) in the United States, the European Commission (EC) and from the Canadian Institute of Research (CIHR) in Canada.

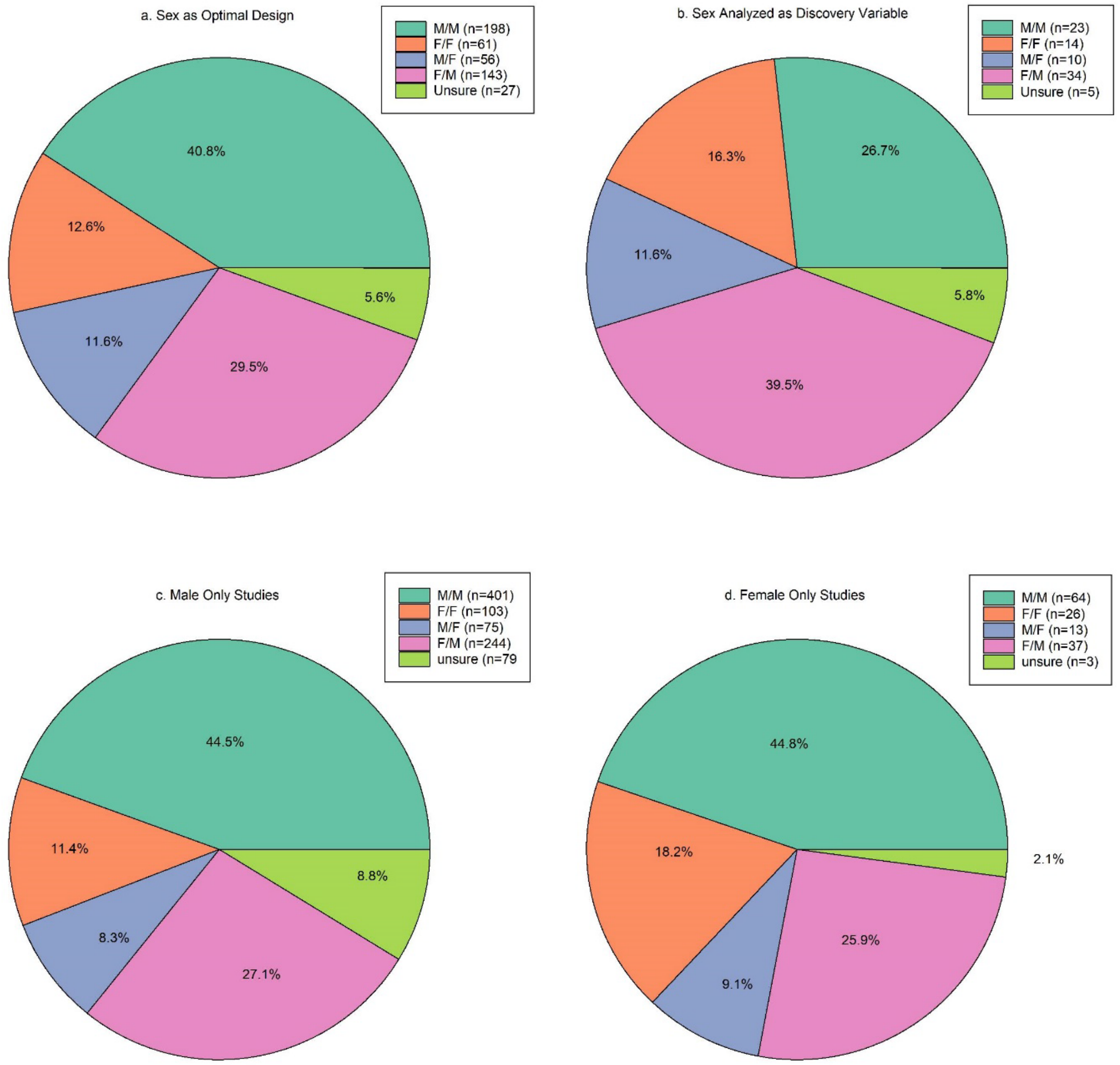

Supplementary Figure 2. Inferred author sex (first author/last author) for papers that reported studies that used an optimal design or analysis for discovery of possible sex differences as well as single sex studies. (a) Distribution of inferred author sex by first or last author of published papers using an optimal design for possible discovery of sex differences (i.e. used equal and consistent number of both sexes throughout study). (b) Inferred author sex by first or last author of published papers that used sex as a discovery variable. Note that M/M percentage is decreased while F/F and F/M are increased compared to a. (c) Inferred author sex by first or last author of published papers that only used male subjects. (d) Inferred author sex by first or last author of published papers in papers that only used female subjects. Note in comparison to male-only papers in (c) that the percentage increased the most for F/F inferred authors for papers that used females-only. F=female, M=male.
