# Supplementary material for: An analysis of neuroscience and psychiatry papers published from 2009 and 2019 outlines opportunities for increasing discovery of sex differences
Source: Nat Commun. 2022 Apr 19;13:2137. doi: 10.1038/s41467-022-29903-3 (PMC9018784; doi:10.1038/s41467-022-29903-3)
Supplement: Supplementary file 3 — Reporting summary [file 41467_2022_29903_MOESM3_ESM.pdf]

nature portfolio

Corresponding author(s): Galea, Liisa

Last updated by author(s): Feb 7, 2022

Reporting Summary

Nature Portfolio wishes to improve the reproducibility of the work that we publish. This form provides structure for consistency and transparency in reporting. For further information on Nature Portfolio policies, see our Editorial Policies and the Editorial Policy Checklist.

Statistics

For all statistical analyses, confirm that the following items are present in the figure legend, table legend, main text, or Methods section.

n/a	Confirmed	
☐	☒	The exact sample size (*n*) for each experimental group/condition, given as a discrete number and unit of measurement
☐	☒	A statement on whether measurements were taken from distinct samples or whether the same sample was measured repeatedly
☐	☒	The statistical test(s) used AND whether they are one- or two-sided<br>*Only common tests should be described solely by name; describe more complex techniques in the Methods section.*
☒	☐	A description of all covariates tested
☐	☒	A description of any assumptions or corrections, such as tests of normality and adjustment for multiple comparisons
☐	☒	A full description of the statistical parameters including central tendency (e.g. means) or other basic estimates (e.g. regression coefficient) AND variation (e.g. standard deviation) or associated estimates of uncertainty (e.g. confidence intervals)
☐	☒	For null hypothesis testing, the test statistic (e.g. *F*, *t*, *r*) with confidence intervals, effect sizes, degrees of freedom and *P* value noted<br>*Give P values as exact values whenever suitable.*
☒	☐	For Bayesian analysis, information on the choice of priors and Markov chain Monte Carlo settings
☒	☐	For hierarchical and complex designs, identification of the appropriate level for tests and full reporting of outcomes
☐	☒	Estimates of effect sizes (e.g. Cohen's *d*, Pearson's *r*), indicating how they were calculated

Our web collection on statistics for biologists contains articles on many of the points above.

Software and code

Policy information about availability of computer code

Data collection	No software was used other than google spreadsheets to collate the data.
Data analysis	Statistica v13 was used.

For manuscripts utilizing custom algorithms or software that are central to the research but not yet described in published literature, software must be made available to editors and reviewers. We strongly encourage code deposition in a community repository (e.g. GitHub). See the Nature Portfolio guidelines for submitting code & software for further information.

Data

Policy information about availability of data

All manuscripts must include a data availability statement. This statement should provide the following information, where applicable:

- Accession codes, unique identifiers, or web links for publicly available datasets
- A description of any restrictions on data availability
- For clinical datasets or third party data, please ensure that the statement adheres to our policy

Source data analyzed during the current study are available in the Dataverse repository, https://doi.org/10.5683/SP3/VDH895.

Field-specific reporting

Life sciences study design

All studies must disclose on these points even when the disclosure is negative.

Sample size	We comprehensively searched across all research papers (excluding reviews and brief communications) published in 6 journals Nature Neuroscience, Neuron, Journal of Neuroscience, Molecular Psychiatry, Biological Psychiatry, Neuropsychopharmacology in 2009 and in 2019. Other studies have either used the same approach of examining all qualified research papers (Will et al., 2017; Mamlouk et al., 2020) or have used the first 20 papers that qualified within the year (Beery and Zucker,, 2011- sample size 841 for 2009; Woitwoitch et al., 2021 - sample size of 720 for 2019 papers). Our sample size of 3191 for two years is similar to Will et al.., 2017 who sampled 4 years and examined 6636 articles or Mamlouk et al., 2020 who examined 1827 papers for one year (2017). As our sample size is predetermined by the number of research papers that are published by each journal each year and these numbers can vary we feel our sample size is appropriate given other papers on this same topic.
Data exclusions	We examined all primary research papers from 2009 and 2019 if the papers used rats, mice, human subjects, fetal cells or cell lines were included. Cell lines included immortalised cell lines, primary cell culture, and stem cell derivatives. As sex of cells matters in a variety of outcomes, papers that used these tissues were included. Non-primary research articles such as reviews and viewpoints were excluded as well as brief communications due to their brevity and following previous literature. Figure 1 shows papers were included or excluded based on our criteria, of the 5649 papers in these journals in 2009 and 2019 we excluded 1759 as they were reviews, viewpoints or brief communications) and a further 697 research articles were not examined as they did not use humans, rats, mice or cell lines as their participants, subjects or tissue. neuroscience and psychiatry domain, as well as the top society journals (Society for Neuroscience, American College of Neuropsychopharmacology) and journals that were chosen in prior research. We used the same exclusion criteria as other published data on this topic (Beery and Zucker, 2011; Will et al., 2017; Mamlouk et al., 2020; Woitwoitch et al., 2021).
Replication	All of the data were obtained from public records and were verified with two independent catalogers (TFS, RKR).
Randomization	As we comprehensively sampled all published papers that fit the criteria across 6 journals (3 journals in each discipline of Neuroscience and Psychiatry) no randomization was done. We chose journals based on the top ISI Clarivate rankings that published primary research papers and subject-specific journals within the neuroscience and psychiatry domain, as well as the top society journals (Society for Neuroscience, American College of Neuropsychopharmacology) and journals that were chosen in prior research.
Blinding	Two data collectors downloaded the data from each journals website (publicly available) onto computers and catalogued the variables into a spreadsheet. Both researchers (RKR, TFLS) were blinded to the hypotheses.

Reporting for specific materials, systems and methods

We require information from authors about some types of materials, experimental systems and methods used in many studies. Here, indicate whether each material, system or method listed is relevant to your study. If you are not sure if a list item applies to your research, read the appropriate section before selecting a response.

Materials & experimental systems

n/a	Involved in the study	
☒	☐	Antibodies
☒	☐	Eukaryotic cell lines
☒	☐	Palaeontology and archaeology
☒	☐	Animals and other organisms
☒	☐	Human research participants
☒	☐	Clinical data
☒	☐	Dual use research of concern

Methods

n/a	Involved in the study	
☒	☐	ChIP-seq
☒	☐	Flow cytometry
☒	☐	MRI-based neuroimaging
